# Left Ventricular Ejection Fraction in Heart Failure—A Parameter to Be Discontinued?

**DOI:** 10.3390/jcm15103646

**Published:** 2026-05-09

**Authors:** Inês Freire, Manuel Vaz da Silva

**Affiliations:** 1 Faculty of Medicine, University of Porto, 4200-319 Porto, Portugal; inesfr2002@gmail.com; 2 Cardiology/Medicine Department, Faculty of Medicine, University of Porto, Centro Hospitalar de S. João, 4200-319 Porto, Portugal

**Keywords:** heart failure, left ventricular ejection fraction, echocardiography

## Abstract

Heart failure (HF) is a multifactorial and heterogeneous syndrome with substantial epidemiological burden, high mortality, and impact on quality of life. In the context of heart failure, left ventricular ejection fraction (LVEF) has been regarded as the most important marker of systolic function and is fundamental in medical research and clinical practice. In research, LVEF has been a major inclusion criterion in most clinical trials over the past few decades. Furthermore, international heart failure guidelines rely on LVEF for the diagnosis of HF and to guide effective treatment. Additionally, our understanding of HF phenotypes and prognosis is mostly grounded in a classification based on LVEF. Nevertheless, there has been a growing debate regarding the role of LVEF in heart failure. In this context, the purpose of this review is to discuss both the advantages and contemporary relevance of LVEF in heart failure, as well as its limitations and controversies. In addition, this review aims to discuss potential alternatives and future directions in heart failure classification, such as new classification methods, alternative measurements of systolic function and imaging techniques, the HLM score, and the use of artificial intelligence and machine learning.

## 1. Introduction

According to the Universal Definition and Classification of Heart Failure [1], a consensus document from several Heart Failure (HF) societies, HF is defined as “a clinical syndrome with symptoms and/or signs caused by a structural and/or functional cardiac abnormality and corroborated by elevated natriuretic peptide levels and/or objective evidence of pulmonary or systemic congestion”. Heart failure is a complex, progressive, multifactorial, and heterogeneous syndrome, known for its high morbidity and mortality, as well as deleterious impact on the quality of life [2,3].

Heart failure (HF) is often considered an epidemic in this century [4] with an estimated prevalence of 1–2% in the adult population [5].

Due to improvements in the treatment of HF and associated comorbidities, as well as the global ageing of the population, there has been an increase in the prevalence of heart failure [3]. On the other hand, incidence increases significantly with age and seems to have stabilised over the past years, with a tendency to decrease in industrialised countries [3]. Despite improvements in the management of this disease and comorbidities, HF still has a high mortality rate and is one of the leading causes of hospital admissions, particularly among older patients [3]. As previously stated, HF represents a substantial economic burden on healthcare systems worldwide, and the associated costs are estimated to increase in the upcoming years [3,5].

LVEF is regarded as the most important marker of cardiac function, being a cornerstone in the context of heart failure research and medical practice. In the context of research, it has been an inclusion criterion in all major clinical trials in recent decades [6,7,8]. The diagnosis, phenotyping, follow-up, prognosis, and treatment are also based on the LVEF measurement according to international heart failure guidelines [9,10,11].

Nevertheless, over the past few years, there has been a growing debate about the use of LVEF in heart failure classification. While some authors emphasise its limitations as the primary phenotyping parameter in HF and call for its urgent replacement [2,12,13]. Other authors highlight all the knowledge that was produced based on LVEF and the lack of a current consensual alternative [6,8].

In this context, this review aims to discuss the advantages and importance of LVEF in heart failure, its limitations, and discuss possible alternatives and future perspectives.

A literature search was conducted between August 2025 and March 2026 in two electronic bibliographic databases (PubMed/MEDLINE and Web of Science). The initial search terms included (but were not restricted to) “heart failure”, “ejection fraction”, “left ventricular ejection fraction”, “classification”, and “limitations”. Original research articles, international guidelines, expert consensus documents, narrative reviews, and editorials were reviewed. Furthermore, references from relevant articles were manually screened for additional pertinent articles.

## 2. The Origin and Definition of Left Ventricular Ejection Fraction (LVEF) in Heart Failure (HF)

In 1962, Folse and Braunwal used a radioisotopic technique to determine the “fraction of left ventricular end-diastolic volume ejected per beat” and demonstrated that it could provide valuable haemodynamic information regarding the left ventricular function [14]. Then, in 1966, Stuart Bartle, a future psychiatrist [7], introduced the term “ejection fraction” to describe the ratio of stroke volume (SV) to left ventricular end-diastolic volume (LVEDV) [15]. This paved the way for the subsequent use of left ventricular ejection fraction (LVEF) in research and clinical practice [16].

The LVEF is expressed in percentage and is defined as the ratio of the stroke volume (the difference between end-diastolic and end-systolic volumes) and the end-diastolic volume (EDV) [17]. LVEF is usually calculated using two-dimensional (2D) echocardiography, although other imaging techniques can be used, such as three-dimensional (3D) echocardiography, cardiac magnetic resonance (CMR), computed tomography (CT), and single-photon emission computed tomography (SPECT) [18]. This parameter varies with age and sex (with women having a normal range higher than men) [19]. Currently, there is no consensus regarding the normal reference values [20,21]. For example, the recommendations from the American Society of Echocardiography and the European Association of Cardiovascular Imaging consider normal values above 52% in men and 54% in women [22], while European and American guidelines consider a normal value at the cutoff of 50% or above, regardless of sex [9,10,11]. It is important to note that the values reported refer to measurements obtained by two-dimensional echocardiography and that the reference values differ across imaging techniques [23].

## 3. Current Relevance and Strengths of Left Ventricular Ejection Fraction (LVEF) in Heart Failure (HF)

In 1992, the SOLVD trial investigators [24] were the first to include LVEF as an inclusion criterion for enrolment in a clinical trial [19,25]. Afterwards, all major clinical trials used LVEF as an inclusion criterion, although the cutoff used for enrolment varied substantially [7,19,25].

**Figure 1:**
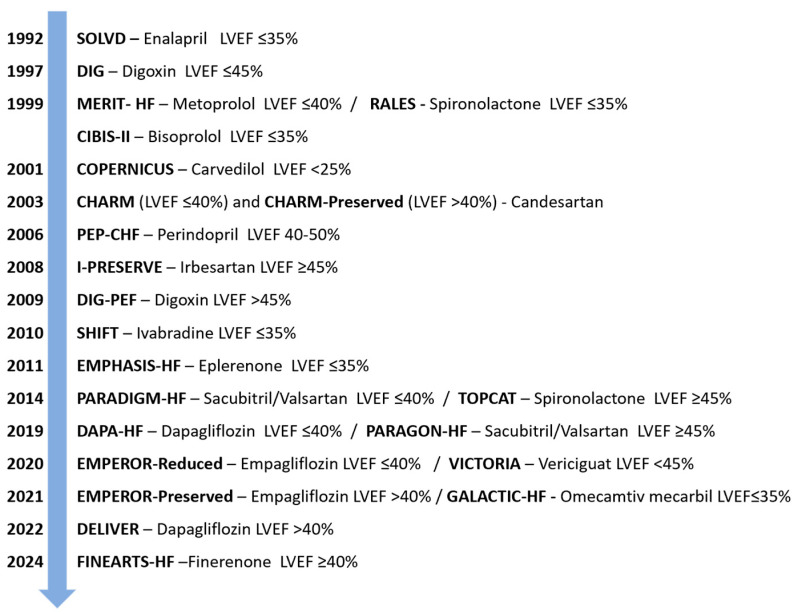
Main clinical trials that used LVEF as an inclusion criterion in the last few decades. SOLVD: Studies of Left Ventricular Dysfunction; DIG: Digitalis Investigation Group; MERIT-HF: Metoprolol CR/XL Randomised Intervention Trial in Congestive Heart Failure; RALES: Randomized Aldactone Evaluation Study; CIBIS-II: Cardiac Insufficiency Bisoprolol Study II; COPERNICUS: Carvedilol Prospective Randomized Cumulative Survival; CHARM: Candesartan in Heart Failure Assessment of Reduction in Mortality and Morbidity; PEP-CHF: Perindopril for Elderly People with Chronic Heart Failure; I-PRESERVE: Irbesartan in Heart Failure with Preserved Ejection Fraction Study; DIG-PEF: Digitalis Investigation Group Preserved Ejection Fraction; SHIFT: Systolic Heart Failure Treatment with the If Inhibitor Ivabradine Trial; EMPHASIS-HF: Eplerenone in Mild Patients Hospitalization and Survival Study in Heart Failure; PARADIGM-HF: Prospective Comparison of ARNI with ACEI to Determine Impact on Global Mortality and Morbidity in Heart Failure; TOPCAT: Treatment of Preserved Cardiac Function Heart Failure with an Aldosterone Antagonist; DAPA-HF: Dapagliflozin and Prevention of Adverse Outcomes in Heart Failure; PARAGON-HF: Prospective Comparison of ARNI with ARB Global Outcomes in HF with Preserved Ejection Fraction; EMPEROR: Empagliflozin Outcome Trial in Patients with Chronic Heart Failure; VICTORIA: Vericiguat Global Study in Subjects with Heart Failure with Reduced Ejection Fraction; GALACTIC-HF: Global Approach to Lowering Adverse Cardiac Outcomes through Improving Contractility in Heart Failure; DELIVER: Dapagliflozin Evaluation to Improve the Lives of Patients with Preserved Ejection Fraction Heart Failure; FINEARTS-HF: Finerenone Trial to Investigate Efficacy and Safety Superior to Placebo in Patients with Heart Failure; Adapted and modified from Dimond et al. (2024) [19].

Some of the main clinical trials that used LVEF as an inclusion criterion, along with their principal results, are summarised in Figure 1 and Table 1.

The introduction of the terms “preserved EF” and “reduced EF” can be traced back to the CHARM (Candesartan in Heart Failure Assessment of Reduction in Mortality and Morbidity) trial in 2003 [12]. In this clinical trial, researchers studied the effects of candesartan in three subgroups: patients with a LVEF > 40% (with preserved EF) and two different subgroups of patients with reduced EF, LVEF ≤ 40% (patients not receiving angiotensin-converting-enzyme inhibitors because of previous intolerance or who were currently receiving angiotensin-converting-enzyme inhibitors) [27]. This nomenclature was later adopted [28] by the 2005 American College of Cardiology/American Heart Association HF Guidelines [29].

The 2016 European Society of Cardiology (ESC) Guidelines proposed a third subgroup, HF with mid-range EF (HFmrEF), when the LVEF is between 40% and 49%, to cover the “grey area” between HFrEF and HFpEF [30]. The purpose of creating this new subgroup was to encourage further research in a population that was often neglected in clinical trials and research [12,30]. Subsequently, in the 2021 ESC Guidelines, this subgroup was renamed HF with mildly reduced EF (keeping the abbreviation) and defined as HF with a LVEF between 41% and 49% [9].

Currently, international guidelines and consensus statements classify HF in three main subtypes: HF with reduced EF (HFrEF) when the LVEF is ≤40%; HF with mildly reduced EF (HFmrEF) when the LVEF is between 41% and 49%, and HF with preserved EF (HFpEF) when the LVEF is ≥50% [1,9,10,11].

More recently, a fourth type of HF has been proposed: HF with improved EF (HFimpEF), although there have been inconsistencies in its nomenclature and definition [1,10,31]. Some authors have also advocated for a fifth type of HF: HF with supranormal EF (HFsnEF) when the LVEF ≥ 65% [32] since some studies reported a worse prognosis in these patients when compared to patients with HPpEF and lower EF values [32,33,34]. For example, in a large clinical cohort of 203,135 patients, the adjusted hazard ratio (HR) for mortality was 1.71 [95% CI 1.64–1.77] when ≥70% [32].

These five subtypes appear to have different phenotypes, responses to treatment, and prognosis [35].

Current heart failure guidelines rely on LVEF not only to diagnose and classify HF, but also to guide treatment and management [9,10,11].

For all these reasons, LVEF has become the central marker of left ventricular cardiac function, with ubiquitous use in heart failure and playing a crucial role in research and clinical practice [4,28,36]. Although it is beyond the scope of this review, LVEF also plays a pivotal role in other areas of cardiology, such as valvular heart disease, myocardial infarction, selection for implantable cardioverter defibrillators, ventricular arrhythmias, and the evaluation of the cardiotoxicity of chemotherapeutic agents [18,23,36,37].

Furthermore, the measurement of LVEF also has several additional strengths that warrant discussion.

A key advantage of LVEF is its universal recognition and acceptance, not only in the field of cardiology but also across the wider medical community [38].

Additional advantages stem from the fact that LVEF is typically calculated using two-dimensional (2D) echocardiography [18]. This imaging modality is widely available, simple to perform, non-invasive, does not use radiation, is inexpensive, and can be rapidly obtained [4,6,39,40]. Furthermore, with the rise of portable echocardiography devices, it is—now more than ever—an evaluation that can be performed at the patient’s bedside [38]. In addition to the LVEF quantification, the use of two-dimensional (2D) echocardiography also provides complementary information regarding the structure and function of the heart [6]. Although normal ranges may vary slightly between sexes, LVEF is independent of other factors such as body weight, height, and race [39,40].

Another advantage of LVEF is that it is considered a good indicator of left ventricular remodelling and eccentric hypertrophy in HFrEF [37]. When there is destruction of cardiomyocytes, the heart undergoes a compensatory mechanism of ventricular remodelling that ultimately leads to the enlargement of the left ventricle in a process called eccentric hypertrophy [12,37]. Despite being maladaptive, this remodelling and enlargement allow an increase in LV capacitance and an increase in the LV end-diastolic volume (LVEDV) [37]. This mechanism enables the maintenance of the stroke volume (SV), even though there is a decrease in myocardial contraction [12]. Since LVEF is calculated as SV divided by end-diastolic volume (EDV) [17], an increase in EDV, with maintenance of SV, results in a decrease in the LVEF, as these variables are inversely related [41]. In these cases, such as in HFrEF, the increase in LVEDV mirrors the decline in systolic function and is measurable as a reduction in LVEF [41]. For this reason, it is considered that the reduction in LVEF is mainly determined by LV geometry and architecture [42].

Additionally, LVEF has been widely used to classify heart failure patients according to phenotypes with different aetiologies and clinical characteristics [6,43,44]. For example, an analysis of the European Society of Cardiology Heart Failure Long-Term Registry (a prospective cohort of 9134 HF patients) [44] and a study based on the Swedish Heart Failure Registry (an ongoing nationwide registry), including 42061 HF patients [45], yielded similar results regarding the clinical characteristics according to LVEF. In both studies, HFrEF patients were younger, more frequently male, and more likely to have an ischaemic aetiology. Conversely, HFpEF patients were older, more frequently female, and more likely to have hypertension, diabetes, or atrial fibrillation [44,45].

Moreover, LVEF has been extensively used for its prognostic value, particularly at lower LVEF values [18,37]. Patients with lower LVEF values are at increased risk of cardiovascular death, hospitalisation, and sudden cardiac death [19]. In a recent study that used the dataset of 33,699 participants from six previous HF randomised controlled trials, the results showed that the incidence of most clinical outcomes increases with a decline in LVEF, with an inflection in the range of 40 to 50%, after which LVEF has less prognostic value [46]. Other studies, focused on the trajectories of LVEF over time, have also demonstrated that a decline in LVEF was associated with higher mortality [47,48].

Finally, guideline-directed medical therapy uses LVEF as a key parameter to distinguish which patients will likely benefit from each approved treatment [9,10,11]. This reflects the fact that the recommendations from guidelines are based on randomised clinical trials that used LVEF as an inclusion criterion over the past decades [28]. Given the methods used in clinical trials, administering therapy to the LVEF phenotypes that showed greater benefit from it is crucial to translate research into everyday clinical practice [8].

For all these reasons, LVEF has become so important and ubiquitous in HF clinical practice and research [4,28,36].

The major strengths of LVEF are summarised in Table 2.

## 4. The Limitations of Left Ventricular Ejection Fraction (LVEF)

Despite all the advantages previously discussed, the use of LVEF has important limitations that deserve discussion, particularly given the ongoing debate about its role in heart failure [2,6,8,12,13].

As previously discussed, the prominence of LVEF largely comes from its use as an inclusion criterion in clinical trials, although cutoffs varied substantially [7,19,25]. However, the definition of LVEF cutoffs in clinical trials was arbitrary and not driven by any physiological distinction [12,49]. In fact, the use of lower LVEF values aimed to improve diagnostic certainty, include patients with worse prognosis, and increase the statistical power of the studies [2,6,8].

Furthermore, LVEF is a continuous variable [19,50] with a normal distribution [9,51]. This unimodal and normal distribution has been shown in recent population-based cohorts [44,52] and previously in one HF clinical trial [53]. In a recent population-based cohort of 9716 new HF patients in Stockholm (between 2005 and 2010), a normal distribution was observed both in the overall study population and in subgroups defined by age and sex [52]. This contrasts with previous studies, mostly using data from hospitalised patients with decompensated HF, that reported a bimodal distribution of LVEF [54,55,56].

Some of the most significant limitations of LVEF are its inter- and intra-observer variability [40,51,52]. It is often considered that this variability can range from 5% to 8% [40,51]. In a recent population-based cohort, it was demonstrated that the standard deviation of the within-person variance in LVEF was 7.4% [52]. Another situation that can lead to bias and affect reproducibility is the fact that the examiner is often unblinded to previous LVEF measurements [52]. Furthermore, there is also considerable inter-modality variability [40,51,57]. In a multicentre randomised trial, aiming to determine the inter-modality variability of LVEF measured by echocardiography, gated single-photon emission computed tomography (SPECT), and cardiovascular magnetic resonance (CMR), in roughly half of the patients, variability exceeded 5%. Bland–Altman analysis demonstrated just a moderate agreement between imaging techniques, with limits of agreement ranging from 28.27% to 35.31%, without significant overestimation or underestimation of LVEF by any modality [57].

Moreover, LVEF follows dynamic trajectories over time [47,48,52]. In a prospective study evaluating LVEF trajectories in HF patients, 2D echocardiography was performed at the beginning of follow-up, after one year, and biennially thereafter, over a 15-year follow-up period. In this study, it was demonstrated that LVEF followed an inverted U-shaped pattern, with a marked increase during the first year, stability for up to a decade, and a gradual decline afterwards (*p* for trajectory < 0.001). Most patients with HFrEF remained in the same category (56%), although a substantial proportion transitioned to HFmrEF (21%) or HFpEF (23%) over time. Conversely, HFmrEF showed marked instability, with patients broadly distributed across categories at the end of follow-up [47]. In another study that included 4943 patients from the Swedish Heart Failure Registry with ≥2 LVEF measurements, approximately one quarter of the patients with HFrEF and HFmrEF had an improvement in EF, whereas a decline was observed in more than one third of the patients with HFpEF and HFmrEF [48]. In a recent population-based cohort, it was shown that the probability of changing category within the first year after diagnosis was substantial [52]. Of note, less than 25% of the patients with HFmrEF remained in the same category after one year [52]. There are several factors that modulate these bidirectional transitions, such as the aetiology, duration of disease, adherence/response to therapy, and other comorbidities [1,39,47]. Some authors argue that this high temporal variability raises concerns about the suitability of using LVEF to guide treatment recommendations [12,52].

A further challenge in this context is digit bias [25]. Digit bias consists of the tendency to round the value to the nearest multiple of 5 when visually assessing LVEF instead of using the precise calculated value [58]. This bias can cause significant imprecision, particularly given that the range of HFmrEF is just 10% [52,59]. A recent study using the European Society of Cardiology (ESC) HF Long-Term registry showed that digit bias in LVEF assessment was substantial, affecting about 37% of the evaluated population [58].

Due to the inter- and intra-observer variability, the dynamic trajectories over time, and the digit bias previously discussed, many authors challenge the use of HFmrEF as an independent phenotype because of its narrow range, high variability over time, and because it was not defined based on a distinct underlying pathophysiology [12,19,47,52,60].

Another limitation of LVEF is that LV geometry is a confounding factor in its assessment [18,61]. The reason for this is that the calculation of LVEF uses only luminal measurements, not taking into account LV geometry [62,63]. According to mathematical models, LVEF can be maintained in the presence of increased wall thickness or reduced LV diameter (leading to reduced end-diastolic volume) despite reduced myocardial contraction [61]. In cases of hypertrophic cardiomyopathy and HFpEF concentric remodelling, although there is a decrease in stroke volume and contractility, EF may remain normal due to the reduction in the ventricular chamber volume that occurs in this type of remodelling [13,64]. In such cases, LVEF may remain normal despite impaired systolic function and reduced stroke volume [64]. Another condition that can alter LVEF is the physiological adaptation and LV remodelling that is observed in endurance athletes [23,40]. Due to the LV enlargement, these athletes frequently have reduced LVEF at rest despite having a normal stroke volume [23,36]. However, during exercise, LVEF becomes supranormal due to haemodynamic changes to meet increasing metabolic demands [40]. This may represent a diagnostic challenge due to its overlapping characteristics with hypertrophic, dilated, and arrhythmogenic right ventricular cardiomyopathies and may complicate interpretation and diagnosis in athletes with underlying heart disease [65,66].

LVEF is usually calculated using two-dimensional (2D) echocardiography, which has inherent limitations [18]. According to the recommendations, LVEF should be evaluated using the “biplane method of disks”, also referred to as the “Modified Simpson’s rule” [22]. This formula uses the geometric assumption that the LV has an ellipsoid shape and regular conformation [18,40] and estimates 3D volumes based on 2D images [36]. However, LV geometry is often distorted in patients with ischaemic heart disease [18,23]. The 2D echocardiography is also highly influenced by image quality [18,36,67] and the acoustic window [4,18], which may be limited by factors such as obesity, lung disease, chest wall deformities, and breast implants [4]. These limitations may lead to a foreshortened ventricle and affect LV volume measurements and therefore LVEF [18,36]. Another potential source of inaccuracy is the endocardial border delimitation [36,68], which can be influenced by poor image quality [64], operator techniques [39,51], lack of standardisation [23], and the definition of papillary muscles [51], all of which can alter the estimation of LV volumes [39]. Even when using semi-automatic delineation techniques, those challenges remain, and there is also inconsistent standardisation and reproducibility [36].

Another limitation is that LVEF is highly influenced by the preload and afterload [39,51,69]. For example, changes in systemic blood pressure, pulmonary venous pressure, or arterial stiffness can result in variations in LVEF [39,51].

Moreover, LVEF is influenced by the heart rate and ventricular synchrony [4,12,64]. Bradycardia may prolong the diastole and increase LV diastolic volume [39], thereby increasing stroke volume and leading to an overestimation of LVEF [51,64]. Conversely, tachycardia reduces the diastolic filling time and LV diastolic volume [39], resulting in a decreased stroke volume and leading to an underestimation of LVEF [51,64]. In the presence of atrial fibrillation (AF), the irregular R-R intervals can also underestimate LVEF [64]. A beat-to-beat variability of 5.8 ± 1.7% has been reported in AF patients [70]. Owing to this variability, in AF patients, LVEF should be calculated as the average of 15 beats—a procedure seldom performed in everyday practice [12]. Abnormal electrical conduction, as seen in left bundle branch block (LBBB) or in patients with a pacemaker, induces ventricular dyssynchrony that can further impair the reliable estimation of LVEF [4].

Additionally, LVEF is influenced by valvular disease, which may lead to either underestimation or overestimation, depending on the type of valvular defect [4,51]. The presence of mitral regurgitation may lead to an overestimation of LVEF [69]. This occurs due to a reduction in preload caused by the regurgitation volume and leads to an increase in the LVEF [64]. In contrast, the presence of aortic stenosis can underestimate LVEF because it causes an increase in the afterload [64], a delay in outflow time peak, and a subsequent reduction in LVEF [40,51].

The assessment of LVEF can be affected by other systemic diseases such as anaemia, thyroid dysfunction, endocrine diseases, infections, metabolic disorders, and other comorbidities [39,40,51]. It can also be conditioned by medications and stress [39]. Similarly, sympathetic activity or vagal stimulation can influence LVEF evaluation [39,40,51]. Some medical conditions can also cause a transient reduction in LVEF with subsequent recovery, such as myocarditis and stress-induced cardiomyopathy (Takotsubo syndrome) [4].

Although LVEF is widely used as a surrogate of systolic function, it is not an accurate measure of myocardial contractility [12,13,18,41]. Because of its mathematical definition, LVEF is inversely related to EDV, which is highly dependent on LV geometry and remodelling [12,38,40,61]. Therefore, architectural changes that modify LVEDV will have a strong impact on LVEF, independently of cardiac contractility, making it a better indicator for remodelling than for assessing systolic function [13,17,39]. In addition, since LVEF is highly dependent on preload and afterload [39,51,69], a correct interpretation of LVEF would require knowledge of LV loads and volumes [13,62]. Furthermore, LVEF does not include any measurement related to the myocardium [23,41,63] and does not consider longitudinal or torsional contraction [39,40,61], nor regional abnormalities [51]. In summary, LVEF serves as an indicator of global ejection performance based on volumetric changes rather than a direct measure of myocardial contraction [18,69,71].

Furthermore, LVEF is not an early marker of systolic dysfunction [18,67]. The LV myocardium is organised into endocardial and epicardial layers, predominantly composed of longitudinal fibres and a mid-myocardial layer mainly consisting of circumferential fibres [67]. Since subendocardial longitudinal fibres are particularly vulnerable to ischaemia, their dysfunction can be compensated by circumferential fibres, allowing LVEF to remain normal [18,67]. However, this subclinical systolic dysfunction can be detected through other techniques, such as echocardiographic strain [72].

A further limitation frequently highlighted is that EF-based phenotypes fail to acknowledge the complexity and heterogeneity of the heart failure syndrome, especially in HFpEF [73,74]. This type of classification ignores the specific aetiology [38] and the presence of important comorbidities [25], both of which are highly relevant for treatment and prognosis [16,25,38]. Moreover, LVEF does not differentiate between systolic and diastolic dysfunction: diastolic impairment is frequent in HFrEF, and HFpEF commonly has subclinical systolic dysfunction that can be identifiable by other imaging techniques [18]. Therefore, a normal LVEF neither excludes nor confirms diastolic dysfunction [38]. Furthermore, HF with a high LVEF is not a benign condition since some studies report worse outcomes in those patients [32,33] with an adjusted hazard ratio (HR) for mortality of 1.71 [95% CI 1.64–1.77] when ≥70% [32].

Additionally, there are pathophysiological mechanisms that are common in HF throughout the spectrum of LVEF, thereby challenging the traditional LVEF-based categorisation [2,7]. Some of these shared mechanisms include endothelial dysfunction, neurohumoral activation (although to different extents), cardiomyocyte injury, myocardial fibrosis, left atrial dysfunction, and ventricular overload [2,7,75].

Another limitation of LVEF is its poor correlation with the patient’s symptoms [18,38]. Despite having preserved EF, HFpEF patients often experience heart failure symptoms, including fatigue, dyspnoea, and reduced exercise tolerance [39].

Furthermore, LVEF has limited prognostic value [53,76,77]. Data from large clinical trials such as CHARM [53] and DIG [76] consistently show that LVEF is a strong predictor of mortality, but only up to 45%. In the CHARM trial, the risk of all-cause mortality rose by 39% for every 10% decrease in LVEF below 45% (HR 1.39; 95% CI 1.32–1.46); however, beyond this threshold, the risk of mortality remained relatively stable despite further increases in LVEF [53]. Similarly, the DIG trial showed that in patients with LVEF ≤ 45%, mortality declined in an almost linear manner across progressively higher LVEF categories, (LVEF < 15%: 51.7%; LVEF 36% to 45%, 25.6%; *p* < 0.0001), by contrast, among patients with LVEF > 45%, mortality rates were similar even after multivariate adjustment (LVEF 46% to 55%: HR 0.92, 95% CI 0.77–1.10; LVEF > 55%: HR 0.88, 95% CI 0.71–1.09) [76]. A similar result was derived from a meta-analysis of the MAGGIC group (comprising 30 studies) that showed that a LVEF above 40% was no longer associated with a risk of mortality [77]. In a population-based cohort, the prevalence of advanced HFpEF (43% of patients) was similar to that of advanced HFrEF (42% of patients) and was associated with poor prognosis and survival, irrespective of LVEF [78]. In a large clinical cohort (with 203,135 patients), it was shown that the adjusted hazard ratios (HR) for mortality had a U-shaped relationship with LVEF with a nadir of risk of 60–65%, a HR of 1.71 (95% CI 1.64–1.77) when ≥70%, and a HR of 1.73 (95% CI 1.66–1.80) at a LVEF of 35–40%. Similar results were observed when analysed by age groups and sex [32]. However, these findings are not consistent across studies. Subsequent research using data from six large randomised trials did not show the described relationship, nor worse outcomes in patients with higher LVEF [51].

For many years, LVEF made the distinction between patients who would benefit from neurohumoral inhibitors (HFrEF) and those without disease-modifying treatments approved (HFpEF) [2,9,79]. However, this paradigm has changed in the last few years. Previous trials had already shown that sodium-glucose co-transporter 2 (SGLT2) inhibitors were effective in the treatment of HFrEF [80]. Recently, the EMPEROR-Preserved trial [81] and DELIVER trial [82] demonstrated that empagliflozin and dapagliflozin, respectively, were effective in the treatment of HFmrEF and HFpEF. As a result, SGLT2 inhibitors are now indicated in HF across the full range of LVEF [10,11]. More recently, the FINEARTS-HF trial demonstrated that the non-steroidal mineralocorticoid receptor antagonist (MRA) finerenone was also effective in the treatment of HFmrEF and HFpEF [26,83]. Therefore, MRAs may be beneficial across the whole spectrum of LVEF, as demonstrated in a recent meta-analysis of the major MRA clinical trials [84]. Additionally, neurohumoral activation is observed throughout the LVEF spectrum despite quantitative differences [2]. This leads some authors to call for the use of these treatments regardless of LVEF [49]. Notably, HF misclassification due to LVEF variability [40,51] and temporal changes [47,48,52] can also lead to delay and undertreatment in certain patients [52,85].

The major limitations of LVEF are summarised in Table 3.

## 5. Possible Alternatives and Future Perspectives

In view of the limitations previously discussed, it is important to review some possible alternatives and future perspectives that have been proposed regarding the assessment of cardiac function in heart failure.

### 5.1. New Perspectives in the HF Classification

Some authors have suggested new LVEF cutoffs for the classification of heart failure [35,60,86]. For example, Romanò proposed in 2025 a simplified classification consisting of “HF with reduced EF” (LVEF ≤ 40%), “HF with below-normal EF” (41% ≤ LVEF ≤ 55%), and “HF with normal EF” (LVEF > 55%) [35]. Additionally, Lam and Solomon have proposed that heart failure should be divided into “HF with reduced EF” (LVEF < 40%), “HF with mildly reduced EF”, and “HF with normal EF” (LVEF ≥ 55% in men and ≥60% in women) in order to acknowledge sex differences and align more closely with normal ranges in the general population [86]. Moreover, Packer proposed a reclassification into “HF with reduced EF” (LVEF ≤ 35%), “HF with mildly reduced EF” (35% < LVEF < 60–65%), and “HF with normal EF” (LVEF ≥ 60–65%) in order to separate different pathophysiological mechanisms, clinical features, and efficacy of neurohormonal antagonists [12,60].

Both a recent clinical consensus statement of the Heart Failure Association (HFA) of the ESC, the Heart Failure Society of America (HFSA), and the Japanese Heart Failure Society (JHFS) [25], and the Universal Definition and Classification of Heart Failure, issued by the HFSA, HFA of the ESC, JHFS, and its writing committee [1], have highlighted the importance of evaluating LVEF trajectories over time rather than relying on a single measurement to guide treatment and to infer prognosis. In this context, the clinical consensus statement from 2025 proposes strategies for optimising treatment over time based on the LVEF trajectory [25]. Furthermore, it has been demonstrated that the prognostic accuracy of LVEF improves when the LVEF is considered as a trajectory over time, as opposed to the use of baseline LVEF alone [87].

Triposkiadis et al. [2] argue that HF should be perceived as a continuous spectrum with overlapping characteristics rather than classified according to rigid categories of LVEF. It is advocated that although the extremes of the spectrum may differ substantially, there are common epidemiological, clinical, and pathophysiological features across the spectrum [2]. The authors believe that this approach better reflects the dynamic and heterogeneous nature of HF and acknowledges the unique trajectory of each patient based on multiple variables [2].

Cikes and Solomon [36] have also proposed another approach to HF assessment. The authors advocated for an integrative approach, incorporating LVEF and additional echocardiographic markers of risk (for example, right ventricular function, left atrial size, and valvular function) and non-imaging parameters, namely clinical parameters (such as age and comorbidities) and biomarkers, to provide a more integrative view of HF and help guide management and prognosis [36].

### 5.2. Myocardial Contraction Fraction (MCF)

An alternative measurement, the Myocardial Contraction Fraction (MCF), was proposed in 2002 [88]. The MCF is defined as the ratio between stroke volume (SV) and myocardial volume (MV) [88] and can be calculated using different imaging techniques [41]. Since myocardial tissue is incompressible, the myocardial volume does not change during systole [88]. Therefore, in this ratio, SV quantifies the degree of myocardial shortening during systole, relative to the overall myocardial volume, despite the fact that the myocardial volume itself remains unchanged [88]. The myocardial volume can be directly measured by 3D echocardiography or can be calculated as the LV mass divided by the myocardial density (1.05 g/mL) [41].

In this context, MCF represents a volume-derived measurement of myocardial shortening that is independent of LV geometry [88]. Furthermore, it is a global contraction index [88], incorporating longitudinal, radial, and circumferential shortening [89]. However, it does not account for the temporal aspects of myocardial shortening [41]. Additionally, MCF is an earlier marker of systolic dysfunction than LVEF in HFpEF, as these patients often exhibit reduced MCF despite normal LVEF [41,90]. MCF is also better at differentiating between physiological and pathological hypertrophy, being reduced in hypertensive hypertrophy and increased in physiological hypertrophy [88]. MCF is a dimensionless ratio and has normal ranges similar to LVEF, which facilitates its use in clinical practice [41,88,91]. However, when calculated using 2D echocardiography, it shares some of the same limitations as LVEF, which are inherent to the imaging technique [41,88]. Therefore, it is recommended to use three-dimensional techniques such as cardiac magnetic resonance (CMR), computed tomography (CT), or three-dimensional (3D) echocardiography, in order to obtain a more precise quantification of stroke volume (SV) and myocardial volume (MV) [41,88]. Moreover, some studies have shown that MCF has greater prognostic value than LVEF [90,92,93]. In a subset of the Framingham Heart Study Offspring cohort, composed of adults without clinical cardiovascular disease and normal LVEF, it was shown that a reduced MCF was associated with an increased risk of major cardiovascular events (over >5-year follow-up), even in individuals with preserved LVEF and after adjustment for cardiovascular risk factors and LV mass [92]. In a recent post hoc analysis of the TOPCAT TRIAL, a decreased MCF was an independent predictor of worse prognosis in patients with HFpEF (HR 0.76, 95%CI 0.64–0.90, *p* = 0.001) [93].

### 5.3. Other Conventional Echocardiographic Parameters

Several other echocardiographic parameters have also been developed and merit a brief mention.

For example, the myocardial performance index (Tei index) aims to combine both systolic and diastolic performance [94] and has demonstrated prognostic value in several types of heart diseases [17,95]. However, it has important limitations, such as being affected by valvular diseases, Valsalva manoeuver, arrhythmias, or ventricular pacing [17].

Another technique worth mentioning is Tissue Doppler Imaging (TDI). TDI is useful to assess systolic and diastolic function and can be used to derive several tissue Doppler indices [96,97]. This technique has shown both diagnostic and prognostic value in numerous heart conditions [96]. This technique is simple, reproducible, and has high temporal resolution [17,96]. Nonetheless, it has important limitations, including angle dependency, requiring high frame rates, and being influenced by heart translation and passive motion of dysfunctional myocardial segments in regional wall motion abnormalities [17,96].

### 5.4. Myocardial Strain and Speckle-Tracking Echocardiography

Another relevant parameter worth discussing is myocardial strain.

Strain is a dimensionless measure of myocardial deformation [64], and it is defined as the percentage of change in myocardial segment length, relative to its baseline length at end-diastole [17]. Conventionally, negative values represent fibre shortening, whereas positive values represent fibre lengthening and thickening [64]. It can also calculate the strain rate, which represents the rate of myocardial deformation [64] and is expressed as a per unit of time (s^−1^) [17]; however, high temporal resolution images are required for its assessment [98].

Strain is normally measured using speckle-tracking echocardiography (STE) [67]. Speckle-tracking echocardiography relies on naturally occurring distinct acoustic markers called “speckles” to track the different regions of the myocardium frame-by-frame during the cardiac cycle [17,64]. The speckles can be tracked in multiple planes and allow measurement of both global and segmental strain, as well as assess LV rotational mechanics [17,64]. This technique has several advantages, such as being angle-independent, not being affected by heart translation, allowing for the assessment of longitudinal, circumferential and radial planes, and having better reproducibility than other imaging techniques [17,64,72]. STE can be applied to 2D echocardiography, which is the most widely used, as well as to 3D echocardiography [72]. The 2D STE has the advantages of superior temporal and spatial resolution [64]. Conversely, 3D STE allows simultaneous multi-plane analysis and a better assessment of rotation, but has a lower resolution than 2D STE [64].

Global longitudinal strain (GLS) represents the average longitudinal strain of 17–18 myocardial segments [17,99], and it is evaluated from apical views [36] at the end of systole [17].

Given that the subendocardium, where the longitudinal fibres are located, is the area most vulnerable to ischaemia [18,61], the subendocardial dysfunction (reflected in GLS) normally precedes the LVEF decline [36,72,100].

In a meta-analysis, normal GLS values ranged from −15.9% to −22.1%, with a mean of −19.7% (95% CI −20.4% to −18.9%) [101]. GLS is usually considered normal at approximately −20% ± 2% [22], although universal reference values have not yet been established [51,98]. When the GLS is >−16%, it is typically considered a sign of myocardial dysfunction [10,72].

The use of GLS has several advantages because it detects subclinical LV dysfunction, is less dependent on LV geometry than LVEF, provides temporal and regional information, and is more reproducible than LVEF [17,64,99]. The use of bull’s eye plots or polar maps is also useful in the distinction of disease-specific strain patterns and regional differences [64,72].

However, GLS also has several limitations, such as the lack of familiarity, high dependence on image quality and frame rates, the presence of variability between vendors, and it can be affected by arrhythmias and respiratory motion [17,64,72,102]. Furthermore, it is influenced by age, sex, and haemodynamic load [72,102] and may require more time for image processing [17].

Nowadays, the assessment of asymptomatic cardiac dysfunction by GLS is already recommended in cardio-oncology guidelines, where a >15% relative reduction in GLS from baseline is considered a sign of cardiotoxicity [98,103].

Since GLS is an earlier marker of subclinical dysfunction, it is particularly useful in HFpEF [61,72,100]. In an echocardiographic sub-study of the TOPCAT trial, impaired longitudinal strain was present in 52% of the patients studied with HFpEF [100]. In a similar sub-study using HFpEF patients from the RELAX trial, 65% of them also exhibited impaired GLS [104]. Furthermore, it is considered a minor echocardiographic criterion for the diagnosis of HFpEF in the diagnostic algorithm proposed in a consensus recommendation from the Heart Failure Association (HFA) of the European Society of Cardiology (ESC) [105].

Furthermore, GLS is a stronger prognostic marker than LVEF [99,100,106]. The echocardiographic sub-study of the TOPCAT trial has also shown that an impaired GLS was independently associated with cardiovascular death and HF hospitalisations [100]. Additionally, a retrospective study demonstrated that GLS is better at predicting all-cause mortality than LVEF over a follow-up period of more than five years and provided additional prognostic information, including in patients with LVEF > 35% [99].

Beyond this, in a large cohort from the general population (Copenhagen City Heart Study), a lower GLS was significantly associated with a higher risk of the composite outcome of incident heart failure, acute myocardial infarction, or cardiovascular death (HR 1.12 [1.08–1.17], *p* < 0.001 per 1% decrease) over a median follow-up of 11 years. This association remained significant after multivariate adjustments for clinical, echocardiographic, and analytical variables [106].

In this context, some authors advocate for the inclusion of GLS as part of the standard echocardiogram, particularly in cases of HFpEF, stage B HF, RV dysfunction, atrial fibrillation, and cardio-oncology [107].

Another aspect worth discussing is the layer-specific myocardial strain, which is considered a promising tool in STE [108,109]. The layer-specific myocardial strain aims to evaluate strain in three different layers (endocardium, mid-myocardium, and epicardium) in order to provide a more comprehensive evaluation of myocardial deformation [109]. In healthy individuals, layer-specific strain progressively decreases from the endocardium towards the epicardium, as well as from the apical region to the base of the left ventricle [109]. This gradient can be explained by the different orientations and distribution of myocardial fibres within the heart [109]. Since many diseases have a heterogeneous effect on the myocardium, this layer-specific approach may enable earlier and more accurate detection of pathological changes [110]. Several studies have demonstrated that this approach could provide incremental value in several conditions [109,110], particularly in ischaemic heart disease [111,112] and cardiomyopathies [113,114]. However, there are no clear reference values for each myocardial layer, and further research is required before this technique can be routinely implemented in clinical practice [109,110].

### 5.5. Global Longitudinal Active Strain Energy Density (GLASED)

Another alternative measure, GLASED (Global Longitudinal Active Strain Energy Density), was proposed in 2022 [63].

To understand this concept, it is important to review another measure, the Myocardial Active Strain Energy Density (MASED), also referred to as “contractance” [62,63]. MASED is derived from the area under the stress–strain curve [115], and it quantifies the energy production per unit of myocardial volume [62] rather than just volumetric output [63]. It is important to note that “stress” measures force per unit area and “strain” measures deformation [62]. Therefore, this measurement combines myocardial strain and wall stress [63], as well as LV geometry and afterload [62]. Furthermore, it is derived from myocardial mechanics [62], rather than from luminal measurements [116].

Global Longitudinal Active Strain Energy Density (GLASED) is the longitudinal form of MASED, and it quantifies the longitudinal mechanical work done per unit volume of myocardium during systole, and it is measured in kJ/m^3^ [63]. GLASED is calculated using four variables: systolic blood pressure, LV wall thickness, LV internal diameter, and global longitudinal strain (GLS) [62,63], which are independent risk markers on their own [62]. The mathematical formula to calculate GLASED is further described in several articles [115,116], and it is a close approximation to the area within the stress–strain curve [62].

Another metric that can be derived from GLASED is the Global Longitudinal Active Strain Energy (GLASE) [63], which represents the total longitudinal work of the LV. This can be calculated by multiplying GLASED by the LV muscle volume [115].

Whereas GLASED reflects myocardial integrity or dysfunction, GLASE indicates whether there is adequate myocardial mass to generate the energy needed for normal LV performance [115] and therefore, is a marker of global LV function [62].

Similar metrics can be calculated for midwall circumferential active strain energy (CASE) and its density (CASED) [63].

These parameters can be obtained from echocardiography or CMR [62].

The main advantages are that GLASED has a solid theoretical basis in engineering physics, accounts for confounders such as LV geometry and afterload, and requires only four measurements [62,63]. It can also be applied to in vivo, in vitro, and ex vivo studies [62,116]. Therefore, it is considered a more informative, holistic, and integrative evaluation of myocardial function and mechanics [62] by assessing the energy production per unit volume of myocardium [63]. Thus, this metric combines the assessment of haemodynamic loading conditions, LV remodelling, and myocardial performance [62].

However, it also has several limitations, such as limited experience in use and familiarity, the need for multiple measurements (which can be time-consuming), the complexity of the calculations, the need for precise geometric measurements (to avoid propagation error), the beat-to-beat variability in arrhythmias, and lack of standardisation [62,115].

To facilitate the application of GLASED in clinical practice, an online calculator has been developed [62].

Several studies have demonstrated that GLASED detects myocardial dysfunction even in the presence of a normal LVEF, as in the case of cardiac amyloidosis [63] and HFpEF [62]. Furthermore, GLASED may have a role in differentiating between physiological and pathological hypertrophy [62].

It has also been demonstrated that GLASED has greater prognostic accuracy than LVEF [63,115]. In a recent low-risk community-based cohort (using the UK Biobank database), GLASED outperformed conventional risk markers in predicting all-cause mortality and major adverse cardiovascular events. GLASED was significantly linked to all-cause mortality and major cardiovascular events, showing the greatest hazard ratio, the best prognostic ranking, and consistent risk discrimination among the three tertiles (*p* ≤ 0.0003) [115].

### 5.6. Other Imaging Techniques

#### 5.6.1. Cardiac Magnetic Resonance (CMR)

Cardiac magnetic resonance (CMR) imaging is considered the gold standard for LVEF measurement due to its high accuracy and reproducibility [23,25,117]. CMR also has the advantage of not relying on geometric assumptions to calculate LVEF, in contrast to echocardiography [36]. Additionally, it is an imaging technique with better spatial and contrast resolution [23].

Furthermore, the use of CMR can provide additional important information regarding myocardial tissue characterisation [10,25,67]. Late gadolinium enhancement (LGE) enables detection and localisation of fibrosis, distinguishing ischemic from non-ischemic aetiologies [10]. T1 mapping also provides a comprehensive evaluation of the myocardial tissue, distinguishing inflammation, oedema, fibrosis, fat or amyloid deposition, and iron overload [25,67]. In this way, CMR imaging techniques can play an important role in determining specific aetiologies [10].

However, this technique is difficult to implement in everyday clinical practice [35] due to its scarce availability, long scan times, and high cost [25]. Additionally, CMR has other limitations, such as its use in patients with claustrophobia or with implantable devices (which may contraindicate CMR or lead to image artefacts), as well as its inability to provide additional haemodynamic information [23,25].

#### 5.6.2. Three-Dimensional (3D) and Contrast-Enhanced Echocardiography

Echocardiography has evolved over the past years, and the more recent echocardiographic techniques, such as three-dimensional (3D) and contrast-enhanced echocardiography, also merit further discussion.

Three-dimensional echocardiography has several advantages when compared with traditional two-dimensional echocardiography [36]. One of the major advantages is that 3D echocardiography does not rely on geometric assumptions when calculating the LVEF because it enables real-time volumetric reconstructions [22,25,36]. Furthermore, it is less affected by LV foreshortening [22]. Several studies have also demonstrated that 3D echocardiography has better accuracy and reproducibility in LVEF assessment than 2D echocardiography when compared with the gold-standard CMR [118,119]. However, when compared with 2D echocardiography, 3D echocardiography has lower spatial and temporal resolution [22,36], as well as longer acquisition times [17].

Contrast-enhanced echocardiography, on the other hand, primarily aims to improve endocardial border delimitation [22,25], an essential step in LVEF measurement [36,68]. Two-dimensional contrast-enhanced echocardiography has been shown to improve accuracy and reproducibility in LVEF measurements [118], especially in cases of suboptimal image quality [22,25,120].

### 5.7. HLM Score

In 2013, a new classification of HF was proposed, the HLM score [121].

This classification is a staging system inspired by the TNM staging used in oncology and aims to provide a comprehensive view of HF [121,122]. Instead of focusing only on the symptoms or LVEF, this score classifies HF according to its multisystemic involvement and has three main components. First, it evaluates the “H” (heart), which reflects the degree of structural and functional damage of the heart (including systolic and diastolic dysfunction, previous myocardial infarctions, and LV remodelling) and is analogous to “T” in the TNM classification. Then, it assesses the “L” (lung), the pulmonary involvement, namely the degree of congestion and pulmonary hypertension. This is analogous to the lymphatic involvement “N” in TNM because of the close anatomical and functional relationship with the heart. Finally, it evaluates the “M” (malfunction of other organs), including renal function (GFR < 60 mL/min), liver function (an increase to at least double the normal level in at least one of the liver parameters AST, ALT, total bilirubin, gamma-glutamyl transferase, or alkaline phosphatase), central nervous system or psychological disorders (using The Beck Depression Inventory, Cardiac Depression Scale, and CT/MRI to exclude stroke), cachexia (>5% oedema-free body weight loss during the previous year or less), and anaemia (haemoglobin < 13 g/dL for men and <12 g/dL for women) or iron deficiency (transferrin saturation < 20% and serum ferritin of 100–299 ng/mL or serum ferritin < 100 ng/mL). Once again, it draws parallels with TNM staging, in this case with “metastasis,” that is, the involvement of other peripheral organs [121,123].

The HLM classification is summarised in Table 4.

This classification has the advantage of providing a holistic framework, integrating clinical, analytical, and imaging parameters, and emphasising the underlying pathophysiological mechanisms of heart failure [67,123]. It may also be a valuable instrument for systematic and structured evaluation of HF progression [121]. The aim of HLM staging is to avoid a simplistic and cardiocentric view of heart failure and acknowledge the importance of multisystemic involvement (with a multivariate model) in order to achieve improved management and prognosis in HF [67,69,122].

Using the HLM classification, the HLM score was created, where “H”, “L”, and “M” are used as numerical variables [123]. This score was created using the linear combination of coefficients derived from the Cox proportional hazards model for composite and individual outcomes, multiplied by 10 and rounded to the nearest whole number. As a result, the HLM score = 2H + 3L + 1M [123]. This HLM score can then be categorised into four HLM stages (HLM-1 to HLM-4) [123].

Several studies have demonstrated that the HLM score can be an important prognostic tool [122,123,124]. In a multicentre prospective cohort (including 1720 hospitalised HF patients), the HLM score demonstrated superior prognostic value than the NYHA classification, ACC/AHA stages, and LVEF assessment in predicting all-cause mortality and HF rehospitalisation at 12 months of follow-up [122]. When comparing the HLM score with LVEF, it demonstrated superior prognostic accuracy, with a higher area under the curve (AUC 0.645 vs. 0.572), which was statistically significant (*p* = 0.002) [122]. Another study has shown that the HLM stage is an independent predictor of CV death (OR: 3.07; 95% CI: 1.54–6.12; *p* = 0.001) and CV death/HF hospitalisation (OR: 2.45; 95% IC: 1.43–4.21; *p* = 0.001) in patients with ischaemic HF [123]. Furthermore, in a study with HF patients with severe aortic stenosis or mitral insufficiency (eligible for transcatheter valve intervention), HLM provided greater prognostic accuracy than the NYHA classification regarding rehospitalisation, with an AUC for HLM = 0.799 vs. NYHA = 0.518, and cardiovascular mortality, with an AUC for HLM = 0.808 vs. NYHA = 0.522 at a 6-month follow-up, and achieving similar results at a 12-month follow-up [124].

### 5.8. Artificial Intelligence (AI)

Artificial intelligence (AI), machine learning (ML), and deep learning (DL) are increasingly used in cardiac imaging, including in LVEF measurements [35,39]. Some studies comparing the use of AI algorithms and the traditional assessment of LVEF have shown that AI algorithms have a similar level of accuracy, with lower variability than a manual assessment conducted by experts [125,126]. Machine learning (ML) can also play an important role in determining one’s prognosis [2,75]. In a sub-study of the PACT-HF (Patient-Centred Care Transitions in HF) trial, researchers used unsupervised ML algorithms to determine clinical phenotypes and examined the relationship between those subgroups and their clinical outcomes. The six subgroups obtained were defined by comorbidities and comprised a broad range of LVEFs. These clinical phenotypes (generated through unsupervised ML) demonstrated greater prognostic value for the selected clinical outcomes (at 6- and 12-month follow-ups) than traditional LVEF-based categories [75]. Therefore, these unsupervised ML models (which rely on unlabelled data) may contribute to improved phenotyping, especially in heterogeneous groups such as HFpEF [35].

## 6. Conclusions

As discussed in this review, LVEF has well-established importance and recognised strengths, as well as limitations and controversies. Although it is unreasonable to expect the replacement of LVEF in the foreseeable future, acknowledging its limitations is crucial to practising more evidence-based medicine. Furthermore, discussion of potential alternatives is also important for a more comprehensive approach that addresses the complexity and nuances of heart failure syndrome. Additionally, some of the discussed alternatives may play an important complementary role in HF and provide incremental value to a classification based solely on LVEF. Among the alternatives discussed, myocardial strain and GLASED appear to be particularly promising candidates for the assessment of systolic function. Furthermore, regarding staging and prognosis, the HLM score also emerges as a potentially valuable tool. Future studies should incorporate these parameters alongside LVEF in order to further evaluate their clinical utility and generate additional evidence that supports their potential future integration into routine clinical practice.

## Figures and Tables

**Table 1:** Effect of pharmacological treatment on cardiovascular mortality or HF hospitalisation across the LVEF spectrum.

Drug	LVEF < 40% HR (95% CI)	LVEF 40–49% HR (95% CI)	LVEF 50–60% HR (95% CI)	LVEF > 60% HR (95% CI)
Beta-blockers	0.74 (0.62–0.88)	0.83 (0.60–1.13)	0.66 (0.38–1.15)	–
Candesartan	0.82 (0.75–0.91)	0.76 (0.61–0.96)	0.95 (0.79–1.14)	–
Spironolactone	0.69 (0.58–0.82)	0.72 (0.50–1.05)	0.85 (0.61–1.18)	0.97 (0.76–1.23)
Sacubitril/valsartan	0.81 (0.69–0.94)	0.89 (0.73–1.10)	0.89 (0.74–1.06)	1.03 (0.80–1.32)
Empagliflozin	0.75 (0.65–0.86)	0.71 (0.57–0.88)	0.80 (0.64–0.99)	0.87 (0.69–1.10)
Dapagliflozin	0.74 (0.65–0.85)	0.87 (0.72–1.04)	0.79 (0.65–0.97)	0.78 (0.62–0.98)
Finerenone	–	0.84 (0.68–1.03)	0.80 (0.66–0.97)	0.94 (0.70–1.25)

HR = Hazard Ratio 95%; CI = 95% Confidence Interval; – = Lack of available data. Adapted and modified from Dimond et al. (2024) [19] and Docherty et al. (2024) [26].

**Table 2:** Advantages of left ventricular ejection fraction (LVEF) in heart failure (HF).

Advantages
Inclusion criterion in all major randomised clinical trials Used in international guidelines for diagnosis, classification, and management Universal recognition and acceptance Wide availability and simplicity of use ^1^ Lack of radiation exposure ^1^ Suitable for bedside assessment ^1^ Independent of body weight, height, and race Reflects LV remodelling and eccentric hypertrophy Separates phenotypes with different characteristics Prognostic value Key determinant for guideline-directed medical therapy

^1^ When performed using two-dimensional (2D) echocardiography.

**Table 3:** Limitations of left ventricular ejection fraction (LVEF) in heart failure (HF).

Limitations
Based on arbitrary cutoffs defined in randomised clinical trials Continuous variable with normal distribution Inter- and intra-observer variability Inter-modality variability Follows dynamic trajectories over time Digit bias Narrow range of HFmrEF LV geometry as a cofounding factor Geometric assumptions and endocardial border delimitation Plane orientation dependency Ignores fibre architecture and rotation Not an accurate measure of systolic function and contractility Not an early marker of systolic dysfunction Load dependent: preload and afterload Dependent on heart rate and ventricular synchrony Influenced by valvular disease Influenced by other systemic diseases/conditions Poor marker for HFpEF and other conditions EF-based phenotypes oversimplify HF, hiding heterogeneity Existence of shared mechanisms across the spectrum Poor correlation with symptoms Limited prognostic value Existence of effective treatments across the EF spectrum

**Table 4:** Simplified HLM classification.

Heart (H)	Lung (L)	Malfunction of Other Organs (M)
H1: Diastolic dysfunction and/or structural damage, without systolic dysfunction (LVEF ≥ 50%) H2: Systolic or diastolic dysfunction accompanied by structural damage but without evidence of LV enlargement. H3: Left or right ventricular systolic dysfunction, or diastolic dysfunction, in the presence of structural cardiac damage and LV remodeling H4: Biventricular systolic dysfunction	L0: Absence of lung involvement L1: Asymptomatic haemodyna- mic congestion L2: Symptomatic lung congestion L3: “Cardiac lung” ^1^	M0: Absence of other organ dysfunctions M1: Single organ dysfunction related to HF ^2^ M2: Existence of two distinct organ dysfunctions related to HF ^2^ M3: Existence of three or more distinct organ dysfunctions related to HF ^2^

^1^ Arterialization of precapillary and postcapillary pulmonary vasculature. ^2^ Excluding the heart and lungs. Adapted and modified from Severino et al. (2023) [122].

## Data Availability

Not applicable.
